# Variation in terpenoids in leaves of *Artemisia annua* grown under different LED spectra resulting in diverse antimalarial activities against *Plasmodium falciparum*

**DOI:** 10.1186/s12870-022-03528-6

**Published:** 2022-03-21

**Authors:** Darunmas Sankhuan, Gamolthip Niramolyanun, Niwat Kangwanrangsan, Masaru Nakano, Kanyaratt Supaibulwatana

**Affiliations:** 1 Department of Biotechnology, Faculty of Science, Mahidol University, 272 Rama VI Road, Ratchathewi District, Bangkok, 10400 Thailand; 2 Department of Pathobiology, Faculty of Science, Mahidol University, 272 Rama VI Road, Ratchathewi District, Bangkok, 10400 Thailand; 3 Faculty of Agriculture, Niigata University, 2-8050, Ikarashi, Niigata, 9502181 Japan

**Keywords:** Plant factory, LED spectrum, Terpenoid, *Artemisia annua* L., *Plasmodium falciparum*

## Abstract

**Background:**

Productivities of bioactive compounds in high-value herbs and medicinal plants are often compromised by uncontrollable environmental parameters. Recent advances in the development of plant factories with artificial lighting (PFAL) have led to improved qualitative and/or quantitative production of bioactive compounds in several medicinal plants. However, information concerning the effect of light qualities on plant pharmaceutical properties is limited. The influence of three different light-emitting diode (LED) spectra on leaf fresh weight (FW), bioactive compound production and bioactivity of *Artemisia annua* L. against the malarial parasite *Plasmodium falciparum* NF54 was investigated. Correlation between the *A. annua* metabolites and antimalarial activity of light-treated plant extracts were also determined.

**Results:**

*Artemisia annua* plants grown under white and blue spectra that intersected at 445 nm exhibited higher leaf FW and increased amounts of artemisinin and artemisinic acid, with enhanced production of several terpenoids displaying a variety of pharmacological activities. Conversely, the red spectrum led to diminished production of bioactive compounds and a distinct metabolite profile compared with other wavelengths. Crude extracts obtained from white and blue spectral treatments exhibited 2 times higher anti-*Plasmodium falciparum* activity than those subjected to the red treatment. Highest bioactivity was 4 times greater than those obtained from greenhouse-grown plants. Hierarchical cluster analysis (HCA) revealed a strong correlation between levels of several terpenoids and antimalarial activity, suggesting that these compounds might be involved in increasing antimalarial activity.

**Conclusions:**

Results demonstrated a strategy to overcome the limitation of *A. annua* cultivation in Bangkok, Thailand. A specific LED spectrum that operated in a PFAL system promoted the accumulation of some useful phytochemicals in *A. annua*, leading to increased antimalarial activity. Therefore, the application of PFAL with appropriate light spectra showed promise as an alternative method for industrial production of *A. annua* or other useful medicinal plants with minimal environmental influence.

**Supplementary Information:**

The online version contains supplementary material available at 10.1186/s12870-022-03528-6.

## Background

*Artemisia annua* L. is a valuable medicinal plant that has been used in China for over 2000 years to treat several infectious diseases [1, 2]. One of the main bioactive compounds from *A. annua* is artemisinin, a sesquiterpene lactone that exerts strong antimalarial activity against various malarial parasites, including the drug-resistant forms of *Plasmodium falciparum* [3]. Artemisinin is best known for its antimalarial activity but it also has anti-adipogenesis, anti-tumor, and antibacterial properties [4]. It is equally cytotoxic against a variety of cancer cells [5, 6]. Another sought after metabolite from *A. annua* is artemisinic acid, a late-stage more stable precursor of artemisinin that can be cost-effectively converted to the latter via chemical synthesis [7, 8].

Multidrug-resistant *P. falciparum* malaria has evolved widely in many countries in tropical areas of sub-Saharan Africa and South-East Asia, where the production of *A. annua* as a source of an antimalarial compound becomes more commercially relevant [9]. The *A. annua* plant originates from China but can grow in a variety of cool temperate and subtropical areas in northern, middle and eastern parts of Asia [10]. Cultivation of *A. annua* requires a large land area, with at least six months from planting to harvest [9]. Artemisinin content from *A. annua* grown in natural habitats ranges between 0.01 and 2% dry weight depending on the genotype, geographic distribution and other environmental conditions [9–13]. A field trial study in Nigeria revealed that seed sources and growing seasons affected leaf biomass and artemisinin production in *A. annua* grown in lowland humid tropical areas. Seeds from China, India and the USA showed poor growth with low artemisinin content, while two non-photoperiodic hybrid lines from Brazil exhibited better growth and higher concentration of artemisinin. The Brazilian hybrid lines exhibited fresh herbage biomass yields of more than 30 tons/ha and produced up to 1.0975% artemisinin [14]. In Thailand, domestic cultivation of *A. annua* and large-scale commercialization of artemisinin production is limited by changes in environmental parameters; plants can be cultivated in northern provinces but show poor growth in hotter areas [15]. Some plants exhibited abnormal characteristics such as stunted growth, premature flowering, abnormal inflorescences, no seed sets and low artemisinin production. These limitations have hampered the large-scale cultivation of *A. annua* for commercial production of artemisinin, and an efficient cultivation platform for *A. annua* is urgently required.

Recently, indoor plant factory systems with artificial lighting (PFAL) have been developed to promote the efficient production of vegetables and medicinal plants [16]. Plant factory systems with artificial lighting offers several benefits over traditional outdoor cultivation such as high yield, reduced pesticide usage and high nutrition content. This system is of interest for research and development, commercial production in industry and urban agriculture. Among PFAL system components, light plays a key role in regulating the growth and development of plants. Pre-harvest treatment of plants with artificial light induced morphology by altering growth characteristics and enhancing biomass production [17–19]. Several studies have demonstrated the specific influence of the light spectrum on the contents of bioactive compounds. For example, soybean sprouts grown under blue light (470 nm) displayed enhanced antioxidant activities along with high contents of total phenolic compounds and total isoflavones [20]. Artichoke seedlings treated with red light (wavelengths between 600 and 700 nm) produced more leaves, while blue light adversely affected leaf growth [21]. Significant increases in total ginsenosides were detected in ginseng roots grown under blue wavelengths of 450 to 470 nm, while growth under other wavelengths (380 and 670 nm) showed no significant differences from roots grown in the dark [22]. Taken together, these findings suggest that specific light spectra promote the production of plant metabolites, which influence the bioactivity of medicinal plants. However, information concerning the effects of artificial light on the pharmaceutical properties of medicinal plants remains limited.

This study applied PFAL under different LED spectra to develop an alternative method for the industrial production of *A. annua* in Thailand. Light-emitting diode (LED) lamps were employed as the light source with the ability to generate tunable and narrow-bandwidth wavelengths. The impact of PFAL under different LED spectra on leaf fresh weight (FW) and bioactive compound production, as well as bioactivities of leaf extracts against the NF54 strain of the malarial parasite *P. falciparum* were investigated. Results will promote the development of strategies to enhance industrial production of plant-derived pharmaceuticals with reproducible quality and productivity.

## Results

### Improvement of leaf fresh weight in PFAL under different LED spectra

**Fig. 1:**
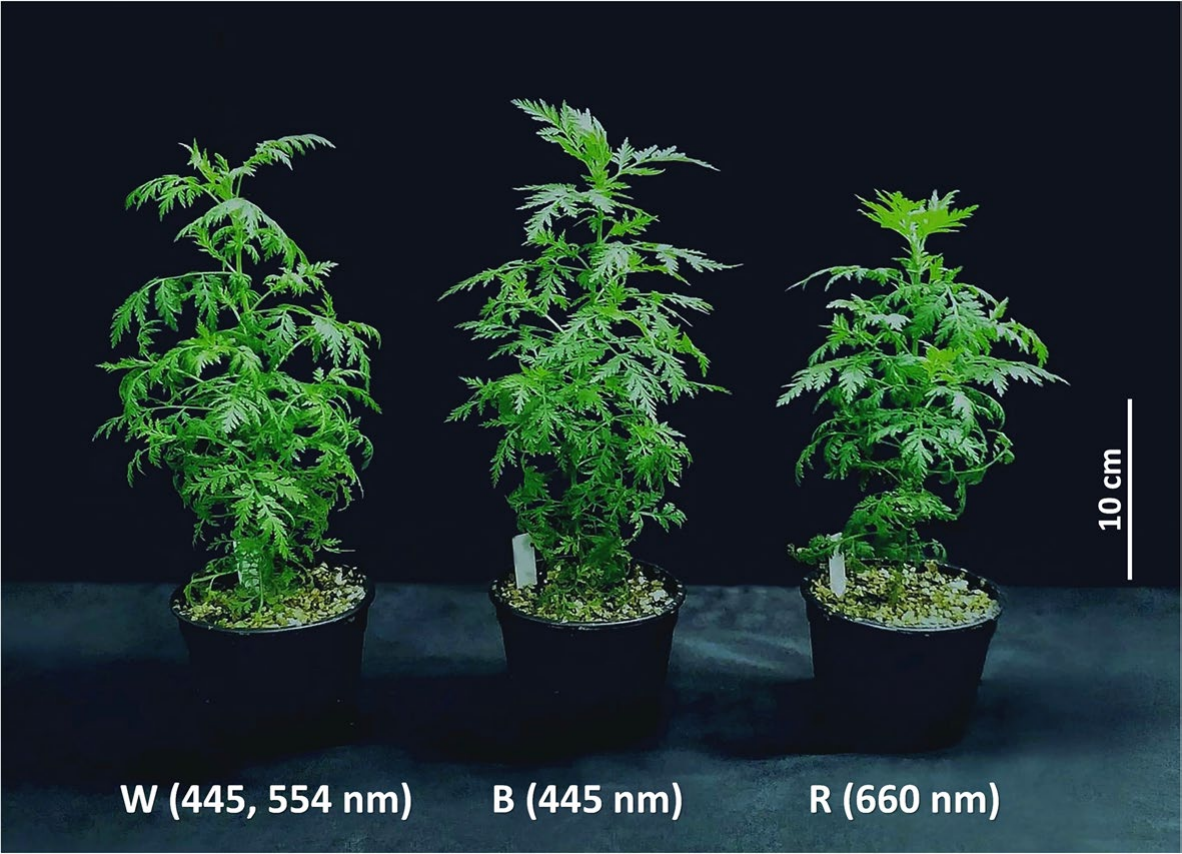
Morphological growth of *A. annua* plants grown indoors. An indoor plant factory with artificial light (PFAL) was set up using different LED spectra as W, white LED light (445, 554 nm), B, blue LED light (445 nm) and R, red LED light (660 nm) at 200 ± 10 μmol m^−2^ s^−1^ PPFD, 16 h photoperiod, 25.0 ± 0.5 °C and 65.0 ± 1.5% RH. The plants were transplanted and incubated for 45 days before exposure to different light spectra for 7 days

In this study, 45-day-old transplants were grown in a greenhouse under natural light, yielding leaf FW of 1.02 g per plant after 7 days (Table S1). In parallel with greenhouse cultivation, *A. annua* plants were grown under PFAL at 25.0 ± 0.5 °C and 65.0 ± 1.5% relative humidity (RH), with photosynthetic photon flux density (PPFD) of 200 ± 10 μmol m^−2^ s^−1^ and 16 h photoperiod. Different LED wavelengths were employed as W, white LED light (λ_max_ = 445, 554 nm), B, blue LED light (λ_max_ = 445 nm) and R, red LED light (λ_max_ = 660 nm). After 7 days, the effects of the LED spectrum on leaf FW were assessed. Under PFAL conditions, plants produced more leaves with higher leaf FW compared with those grown in a greenhouse under natural light conditions. A bushy characteristic was observed in all PFAL plants (Fig. 1). Plants grown under white spectrum showed highest leaf FW of 1.91 g per plant (Table 1). Statistical analyses indicated no significant influence of blue and red light on leaf FW.

**Table 1:** Leaf fresh weight, content and yield of artemisinin and artemisinic acid from leaf extracts of *A. annua* grown under different light spectra for 7 days

Treatment	Leaf FW (g)	Content (mg mL^−1^ dry weight)		Yield (mg plant^−1^)	
		Artemisinin	Artemisinic acid	Artemisinin	Artemisinic acid
W (445, 554 nm)	1.91 ± 0.08 ^a^	0.45 ± 0.01 ^a^	0.29 ± 0.01 ^b^	0.87 ± 0.04 ^a^	0.55 ± 0.02 ^a^
B (445 nm)	1.66 ± 0.05 ^b^	0.37 ± 0.01 ^b^	0.35 ± 0.01 ^a^	0.62 ± 0.01 ^b^	0.58 ± 0.03 ^a^
R (660 nm)	1.59 ± 0.05 ^b^	0.36 ± 0.00 ^b^	0.25 ± 0.01 ^c^	0.57 ± 0.02 ^c^	0.39 ± 0.01 ^b^

Different letters within the same column indicate highly significant differences at *p* ≤ 0.01 of mean (± SD) by Tukey’s HSD test

### Increasing productivity of artemisinin and artemisinic acid under LED light treatments

After 7 days of light treatment, leaf samples were harvested at the 3rd to 7th positions from the shoot apex to determine the levels of artemisinin and artemisinic acid. Highest artemisinin content (0.45 mg mL^−1^ dry weight) was observed in plants grown under white spectrum, while those grown under blue and red spectra exhibited the same level of artemisinin (Table 1). By contrast, artemisinic acid was most abundant in plants grown under blue light treatment (0.35 mg mL^−1^ dry weight), followed by white and red spectra, respectively. High productivities of artemisinin and artemisinic acid were obtained from white and blue light treatments, while red treatment gave lower amounts of both compounds. High artemisinin content was also reported in leaves of *A. annua* grown under greenhouse conditions, probably due to temperature-induced stress (Table S1); however, lower leaf FW indicated that productivities were significantly less than those grown under PFAL.

### LED spectra triggered specific regulation on phytometabolite production

Having verified that artificial light enhanced artemisinin and artemisinic acid production, gas chromatography-mass spectrometry (GC-MS) was used to investigate how other volatile compounds in leaf extracts were affected by different light spectra. Results revealed 62 volatile compounds with more than 70% match quality to the mass spectral library (Wiley 7 no. 1). Most of the detected metabolites were non-terpenoids, while the rest were categorized as terpenoids consisting of 7 monoterpenes, 11 sesquiterpenes, 3 diterpenes and 9 triterpenes (Table S2). Highest content proportion of the detected terpenoids was sesquiterpenes (41–43%), followed by triterpenes (24–28%), diterpenes (16–19%) and monoterpenes (10–16%) (Fig. S1). Among these compounds, two sesquiterpenes as germacrene-D and trans-β-farnesene showed significant increases in abundance. Germacrene-D was produced under white spectrum at 120% relative to the internal standard, while trans-β-farnesene was produced at 104.1% relative to the standard under red LED treatment. By contrast, a preliminary study revealed that the terpenoid profile of greenhouse-grown plants exhibited different proportions of terpenoid compounds as 33% triterpenes, 29% sesquiterpenes, 28% diterpenes and 10% monoterpenes. Diterpene phytol content was high (71.1%), while the remaining metabolites were detected at low levels compared with plants grown under the indoor system.

### Diverse anti-*Plasmodium falciparum* activity of crude extracts derived from different light treatments

**Fig. 2:**
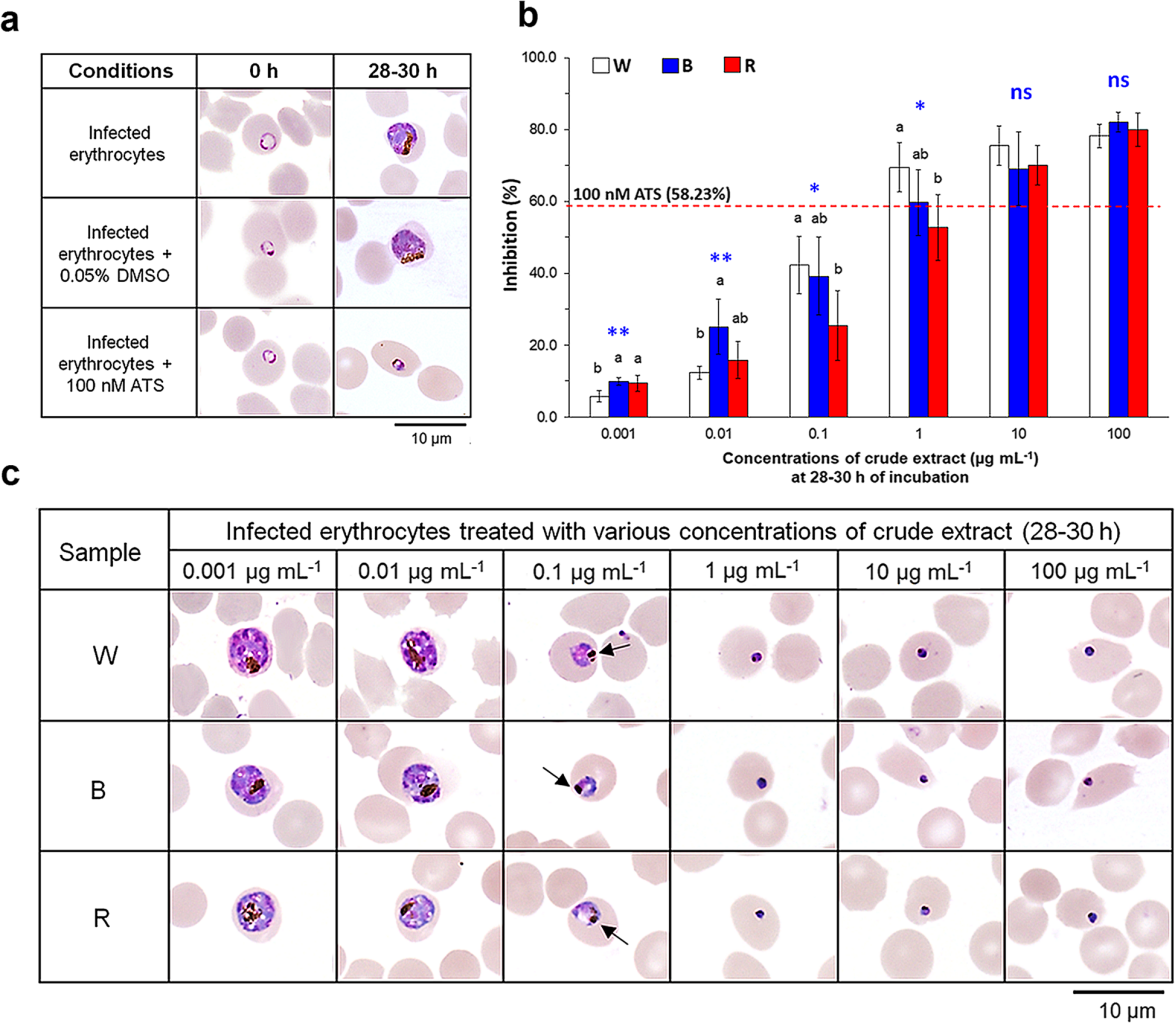
Inhibition of the multidrug-resistant parasite *Plasmodium falciparum* (NF54) using leaf extracts of *A. annua* grown under different artificial light treatments. Microscopic assay of NF54 growth was observed after 0 and 28–30 h of incubation under three conditions. Medium containing 0.05% DMSO and medium containing 100 nM artesunate (ATS) were used as the negative and positive controls, respectively (**a**). Growth inhibition (%) of NF54 after 28–30 h of incubation with six concentrations (0.001–100 μg mL^−1^) of leaf extracts (**b**). Crude extracts were obtained from *A. annua* leaves grown under different LED spectra as W, white LED light (445, 554 nm), B, blue LED light (445 nm) and R, red LED light (660 nm). Other parameters were kept constant as 200 ± 10 μmol m^−2^ s^−1^ PPFD, 16 h photoperiod, 25.0 ± 0.5 °C and 65.0 ± 1.5% RH. Different letters in each group of bars indicate significant difference at *p* ≤ 0.05 (*) or highly significant difference at *p* ≤ 0.01 (**) analyzed by Tukey’s HSD test. Error bars represent standard deviation. Microscopic inspection of NF54 further confirmed that inhibition was concentration-dependent (**c**), in agreement with results shown in **b**

*Artemisia annua* has been determined as the best source of antimalarial compounds. Therefore, an *in vitro* anti-*P. falciparum* assay was performed to investigate the bioactivity of plants grown under different light spectra. The leaf extracts of each light treatment were dissolved in 0.05% dimethyl sulfoxide (DMSO) and added to the parasite at concentrations ranging between 0.001 and 100 μg mL^−1^. Artesunate (ATS), a semi-synthetic derivative of artemisinin recommended by the World Health Organization (WHO) for treatment of patients with *P. falciparum* malaria [23], was dissolved in 0.05% DMSO to a final concentration of 100 nM and used as the positive control. The solution containing DMSO alone was used as the negative control. At 0 h the parasite was in the ring form, as revealed by light microscopy (Fig. 2a). Infected erythrocytes treated with DMSO developed into schizonts after 28–30 h of incubation, indicating that DMSO alone did not inhibit the parasite. Artesunate treatment, on the other hand, arrested parasite growth at the ring stage even after 28–30 h of incubation. Crude extracts were also capable of inhibiting *P. falciparum* but the observed effect depended on the light spectrum and concentration of the extracts (Fig. 2b). At 1 μg mL^−1^, all crude extracts significantly inhibited growth of *P. falciparum*. Crude W showed the highest inhibitory percentage, followed by crude B and crude R, respectively. Interestingly, both crude W and B exhibited higher inhibitory activities than 100 nM ATS. Further increasing extract concentration beyond 1 μg mL^−1^ resulted in higher inhibition by all three extracts, although the difference between extracts was no longer significant.

Probit analysis was performed to quantify the inhibitory effect of plant extracts. Obtained IC_50_ values were 0.51, 0.52 and 1.11 μg mL^−1^ for crude extracts W, B and R, respectively suggesting that *A. annua* was potent even in a crude form. Significant antimalarial activity (IC_50_ 2.23 μg mL^−1^) was also found in the leaf extract of greenhouse-grown plants, though at 4 times less than plants grown under white and blue spectra. This result suggested that a judicious choice of artificial light spectra improved the bioactivity of plant-derived crude extracts.

Growth inhibition by plant extracts was further validated using light microscopy. Similar to the *in vitro* inhibition assay, all crude extracts showed concentration dependence in their inhibitory activities (Fig. 2c). Normal schizonts were detected in samples treated with low extract concentrations (0.001 and 0.01 μg mL^−1^), while increasing the concentration to 0.1 μg mL^−1^ caused hemozoin clumping. Treatment with 1 μg mL^−1^ crude extracts completely inhibited the parasite, as evidenced by the presence of dead ring stage parasites. Finally, at concentrations of 10 and 100 μg mL^−1^, inhibition of parasite growth was not significantly different among all crude extracts. The DMSO-treated sample showed no parasitic inhibition under light microscope, suggesting that inhibitory abilities were derived from the crude extracts.

### Correlation between abundance of plant metabolites and anti-*Plasmodium falciparum* activity in response to different light conditions

**Fig. 3:**
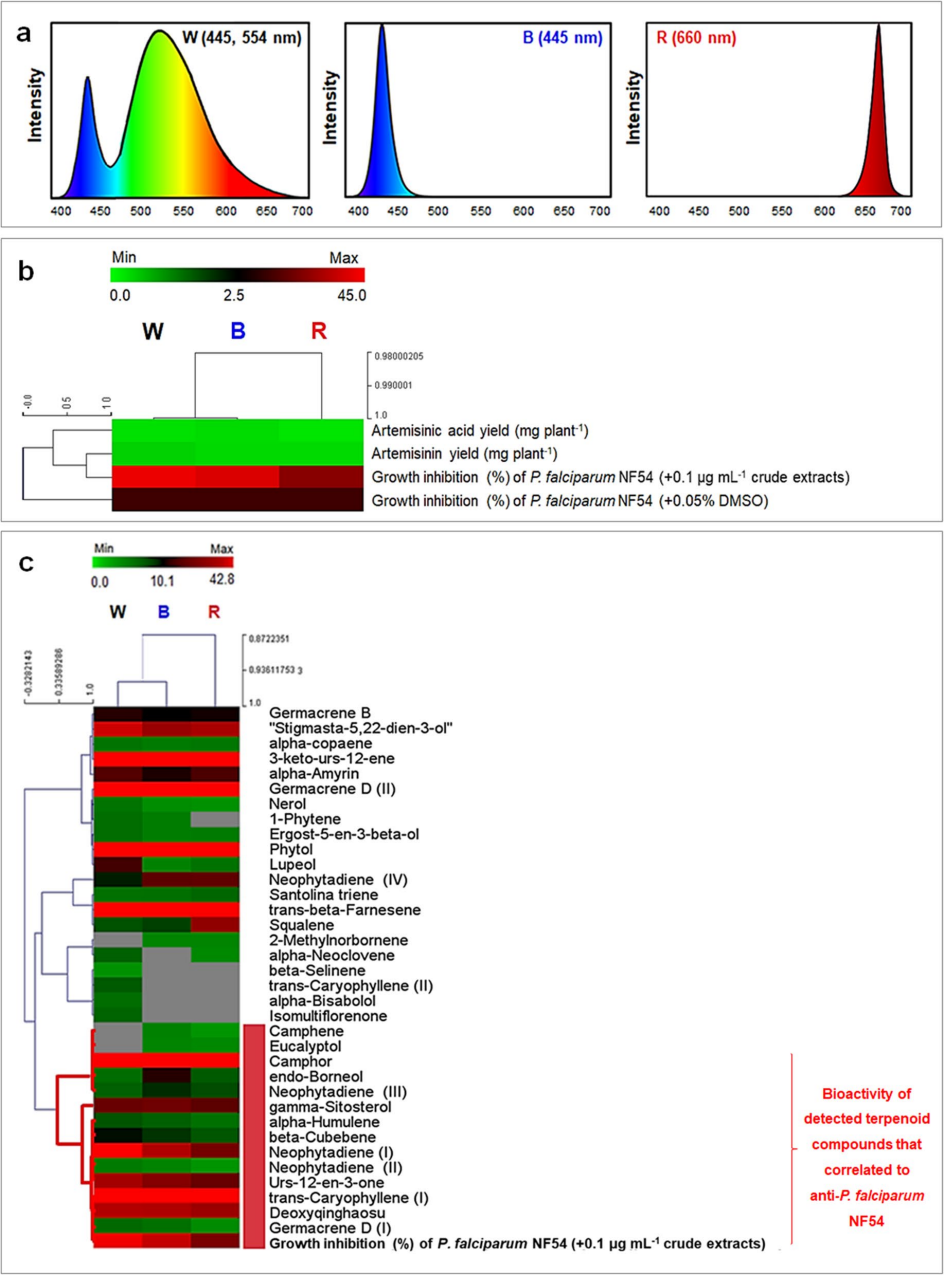
Variations in terpenoid profiles among *A. annua* grown under different LED spectra at 200 ± 10 μmol m^−2^ s^−1^ PPFD, 16 h photoperiod, 25.0 ± 0.5 °C and 65.0 ± 1.5% RH. Spectral profiles of light treatments (**a**) as (W, white LED light (445, 554 nm), B, blue LED light (445 nm) and R, red LED light (660 nm). HCA of artemisinin and artemisinic acid yields (mg plant^−1^) with growth inhibition (%) of NF54 (**b**). HCA of relative abundance of terpenoid compounds and antimalarial activity toward *P. falciparum* NF54 (**c**). Green color indicates low amounts of compounds and low inhibitory activity in each treatment, whereas those with high amounts or high inhibitory activities are colored red. Shorter branch length indicates a higher similarity between the two datasets. Strongly correlated terpenoids with antimalarial activities are labeled with a red bar

Hierarchical clustering analysis (HCA) based on the Pearson correlation was performed to explore the relationship between the abundance of various *A. annua* metabolites and anti-*P. falciparum* activity of the leaf extracts. Datasets used in the first HCA consisted of the abundance of artemisinin and artemisinic acid from different light treatments, and percentage inhibition of *P. falciparum* by *A. annua* extracts derived from different light treatments. Results showed that yields of artemisinin and artemisinic acid highly correlated with extract activities against *P. falciparum* NF54 (Fig. 3b). Data from the white and blue light experiments were also strongly clustered, confirming that plants grown under these spectra exhibited similar responses in plant metabolite production, probably because of the overlapped spectral profiles at 445 nm. The properties of LED lamps are depending on designed spectrum, Fig. 3a revealed the blended wavelengths of 445 and 554 nm that visually present in white LED (W). To extend the scope of the analysis, another round of HCA was performed focusing on other terpenoid compounds. Results showed that data from the white and blue light treatments were similarly clustered, with those from the red light treatment forming an outgroup. The abundances of twenty terpenoids, comprising 4 monoterpenes (camphene, eucalyptol, camphor and endo-borneol), 5 sesquiterpenes (alpha-humulene, beta-cubebene, trans-caryophyllene, germacrene-D and deoxyqinghaosu), a diterpene (neophytadiene) and 2 triterpenes (gamma-sitosterol and urs-12-en-3-one) were strongly correlated with antimalarial activity (Fig. 3c).

## Discussion

High incidences of malaria infections as well as widespread multidrug-resistant *P. falciparum* malaria, especially in Northeastern Thailand, have increased the demand for artemisinin and its associated derivatives [24]. However, cultivation of *A. annua* in Thailand is limited by several environmental factors including high humidity and high temperature. Our preliminary study revealed low leaf FW and leaf wilting in *A. annua* grown in a greenhouse under natural light at high uncontrolled temperature. The temperature in the greenhouse during the experiment was recorded as 38.5 ± 0.5 °C, which was 13–15 °C higher than the optimal growth temperature of *A. annua* suggested by the WHO monograph [9]. Temperature is one of the factors that hamper *A. annua* cultivation for large-scale production. By contrast, *A. annua* grown under the PFAL system exhibited bushy characteristics, resulting in higher leaf FW. Maximum leaf FW was obtained from white spectral treatment at 1.87 times greater than FW of greenhouse-grown plants, suggesting a positive response of *A. annua* to white LED spectrum. LED spectra in an environmentally controlled system have been proven to regulate plant productivity. Fresh weight and dry weight of lettuce significantly increased under red LED light [25], blue spectrum induced biomass of amaranth and turnip greens [26] and white light promoted leaf, stem, root growth and overall growth rate of spinach [27].

Different light qualities induced distinct profiles of plant metabolites, and the effect of light spectra was further investigated on the productions of artemisinin and artemisinic acid. Artemisinic acid was chosen for analysis in addition to artemisinin because the plant materials were harvested at a relatively young stage (two months old) when glandular secretory trichomes (GSTs) responsible for producing artemisinin were not fully developed [28, 29]. GSTs matured as the leaves matured but leaves in the lower position remained shaded. Thus, to certify the explicit impact of artificial light, the plants were subjected to top-down LED lighting. Fully expanded leaves at the 3rd to 7th positions from the shoot apex were used for analyses. As expected, *A. annua* grown under a controlled environment with LED light spectra accumulated higher artemisinin and artemisinic acid contents compared with plants grown under natural conditions. White and blue light irradiation notably increased the content of these compounds, compared with red light. *A. annua* grown in its natural habitat produced 0.1–20 mg mL^−1^ dry weight of artemisinin, while highest yields of artemisinin (0.45 mg mL^−1^) and artemisinic acid (0.35 mg mL^−1^) obtained from this experiment were low, possibly due to the young stage of plant materials used.

Improvements of bioactive compounds of *A. annua* under artificial light spectra were recently reported. Lopes et al. [30] revealed increases in artemisinin content in *A. annua* when exposed to different light spectra. They determined that the increased content was induced by artificial light activation of the *ADS* gene in the cyclization steps of regulating transcriptomic artemisinin production. Attempts to promote higher-yielding artemisinin production have also included the selection of high-producing cultivars, use of fertilizers and bio-stimulants, use of phytohormones, use of other environmental stresses such as heavy metals, cold, water deficit and salinity [14, 31–43]. Genetic manipulations of *A. annua* plants have also been widely applied to increase artemisinin content, either by overexpression of endogenous genes and related transcription factors or by suppressing genes in competitive pathways [44–50].

LED light spectra have been shown to affect leaf FW, bioactive compound productivities, and also the production of other plant metabolites. Variations in terpenoid proportions were observed among plants grown under PFAL and those in the greenhouse. Sesquiterpenes were the most abundant terpenoids in leaf extracts of LED light-treated plants, while triterpenes were the highest proportion in greenhouse-grown plants. Interestingly, phytochemical profiles obtained from white and blue spectra were similar due to intersection of the spectral profiles at 445 nm. Previous studies demonstrated that different light qualities impacted terpenoid variability. Red spectrum enhanced abundances of most sesquiterpenes, while blue spectrum induced greater relative abundance of monoterpenes [30]. A similar result was observed in tomato leaf, with monoterpene α-pinene more abundant under blue spectrum [51]. In addition to light treatment, alterations of terpenoid composition and content in *A. annua* were reported by T-DNA insertions at different positions of the host genome [52]. A similar finding was demonstrated by overexpression of the gibberellin biosynthetic gene, as the proportion of monoterpenes in transgenic plants slightly increased compared with the proportion in wild type [37].

Terpenoids are the largest and the most diverse group of plant secondary compounds and they exhibit wide-ranging pharmacological properties [53, 54]. Thus, alteration of terpenoid content and/or composition affects the pharmaceutical properties of medicinal plants. Several studies have focused on the synergistic effect of artemisinin with other terpenoid compounds against malarial parasites. A transgenic line with high squalene content but moderate level of artemisinin was reported to exhibit higher antimalarial activity than an artemisinin-rich line, suggesting that squalene was involved in increasing the bioactivity of crude compounds [52]. Thus, variations of terpenoid compounds in *A. annua* plants under different light conditions should be further considered for pharmaceutical evaluation. Our results revealed that leaf extracts of *A. annua* grown under white and blue spectra exhibited 4 times greater anti-*P. falciparum* activity than plants grown in the greenhouse. HCA revealed compounds that might be involved in increasing the bioactivity of LED-treated plants, with many previously reported to confer various biological activities that benefitted health. For example, the monoterpene camphor, which was abundant across all light conditions in this study, possessed insecticidal, antimicrobial, antiviral, anticoccidial, antinociceptive, anticancer and antitussive activities [55], while caryophyllene, a sesquiterpene, had anti-inflammatory, anticancer, anticarcinogenic, antimicrobial, antioxidative and analgesic properties [56–59]. Caryophyllene significantly inhibited *P. falciparum* in both *in vitro* and *in vivo* assays [60, 61], corroborating our HCA result. The diterpene neophytadiene, abundantly produced in white and blue light treatments, was reported to exert strong bactericidal, antifungal, antipyretic, analgesic, antioxidant and anti-inflammatory activities, while also showing antimalarial properties [62–64]. The triterpenes gamma-sitosterol and urs-12-en-3-one (β-amyrone) were also reported to have anti-inflammatory activity [65–67]. The collective increase of these terpenoid compounds confirmed the potential of using specific LED spectra to promote the bioactivity of *A. annua*.

## Conclusions

In South-East Asia and sub-Saharan Africa, geographical conditions are not suitable for natural production of *A. annua*, and populations suffer from rollback and drug-resistant malaria that cause acute, chronic malarial disease every year. However, production of artemisinin-raw material from *A. annua* plants is limited by the selection of high-producing lines for each specific production area [14, 15]. This study presented a strategy for *A. annua* cultivation in Thailand using a controlled environment supplemented with artificial lighting. Our study showed that specific wavelength of 445 nm, present in white and blue spectra exhibited positive influences on increasing leaf FW over the red spectrum wavelength of 660 nm. The blue spectrum (400–500 nm) was previously reported to promote stomatal conductance, chlorophyll (Chl) a/b and photosystem (PS) activity expression of genes related to the Calvin cycle as well as activate electron flow between PSII and PSI, leading to improved photosynthetic performance and higher biomass production [68, 69]. Light signals promote the contents of artemisinin and artemisinic acid by regulating expressions of the biosynthetic genes involved in artemisinin production as well as suppressing competitive pathways [23, 30]. Artemisinin possesses antimalarial activity but when used alone is less effective to treat chronic malaria and inhibit drug-resistant parasites. Therefore, a co-promoting compound is required to improve antimalarial activity and achieve rapid killing against drug-resistant parasites. Plants exposed to this wavelength also produced higher contents of sesquiterpene caryophyllene and diterpene neophytadiene at about 1.5 fold higher than during red treatment. Caryophyllene and neophytadiene showed effectiveness against *Plasmodium* parasites [60, 61, 64] and were strongly correlated with growth inhibition of *P. falciparum* NF 54, as shown by our clustering analysis. Based on these findings, the specific wavelength of 445 nm promoted leaf FW, as well as plant metabolite production, with positive effects on anti-*P. falciparum* activity of *A. annua.* Accordingly, application of artificial light in Thai plant factories could be implemented as an alternative production system. Results indicated the noteworthy impact of the LED spectrum on controlling productivity and quality of useful bioactive compounds for pharmacological studies.

## Materials and methods

### Plant material selection and preparation

*Artemisia annua* seeds were kindly provided by Dr. Chalermpol Kirdmanee (BIOTEC, NSTDA) since 2000, under the cooperative research project between Mahidol University and the National Center for Genetic Engineering and Biotechnology (BIOTEC), National Science and Technology Development Agency (NSTDA), Ministry of Science and Technology, Thailand. The seeds were germinated and multiplied in the tissue culture lab at the Faculty of Science, Mahidol University before transplanting to *ex vitro* pot plants in the nursery. *A. annua* plants that showed vigorous growth were then selected and transferred to experimental fields in Chiang Rai and Kanchanaburi Provinces to investigate the effect of cultivation conditions on growth and artemisinin production [15]. After four generations of cultivation and selection in the experimental fields, seed-derived plant lines grown in Northern Thailand exhibited better growth parameters compared with those grown at the Kanchanaburi site. Among these, the material of *A. annua* used in this present study is line no. 500 that was developed by our laboratory and subsequently multiplied in the semi-closed system under photoautotrophic condition, so there was no permission needed for the collection of this line. It showed abundant leaves and adequate numbers of fertile seeds after 7–8 months of cultivation. However, this line is sensitive to hot climates and was maintained as seed and *in vitro* cultures in our lab for study usage. Banyai et al. [70] reported artemisinin content of 24 mg g^−1^ dry weight of this line after 50 to 60 days in a controlled growth cabinet, while a tetraploid line (no. 5GC) derived from line no. 500 showed higher artemisinin content (34 mg g^−1^ dry weight) than its original. However, 5GC exhibited lower leaf biomass than no. 500. Under conditions of 50 to 60 days of pot plant growth in a growth cabinet, most of the tested seed-derived *A. annua* clones showed low artemisinin contents (less than 5 mg g^−1^ dry weight). Thus, line 500 was selected for use in our experiments.

*In vitro* plantlets of *A. annua* line no. 500 were maintained and multiplied on 0.7% agar-solidified full-strength MS medium [71] supplemented with 3% (w/v) sucrose before incubation at 25 ± 2 °C, 60 ± 5% relative humidity (RH) and 16 h photoperiod with 60 ± 5 μmol m^−2^ s^−1^ photosynthetic photon flux density (PPFD). *In vitro* plantlets with well-performed shoot and root growth were transferred to semi-closed vessels, each with a 0.45 μm membrane filter to allow air ventilation. Vermiculite was used as the supporting material, supplemented with sugar-free MS liquid medium. The plantlets were acclimated for 15 days under the same conditions before transfer to *ex vitro* conditions in pots containing vermiculite and then incubated under 25 ± 2 °C, 65 ± 2%RH, 16 h photoperiod with 100 ± 5 μmol m^−2^ s^−1^ PPFD fluorescent lamps.

### Cultivation of *A. annua* under greenhouse conditions

*A. annua* pot plants (45-day-old transplants) that exhibited similar growth performances of shoots, leaves and branching were selected for growth in a greenhouse under natural light. Average microclimate conditions between 17 and 23 January 2019 were recorded as 38.5 ± 0.5 °C and 59.4 ± 1.7% RH. According to the Meteorological Department of Thailand and the Thai Astronomical Society, average light intensity was 1,045 ± 35 μmol m^−2^ s^−1^ with 11.4 h day^−1^ photoperiod during these dates. After 7 days, expanded leaves at the 3rd to 7th positions from the shoot apex were excised from the clump. The leaf samples were kept at −80 °C for further analyses.

### LED spectral treatments and conditions of PFAL

Pot plants of *A. annua* (45-day-old transplants) with good growth performances were transferred to the culture shelves and exposed for 7 days under different LED spectra (Growlab Agritech Co., Ltd., Thailand) as W, white LED light (λ_max_ = 445, 554 nm), B, blue LED light (λ_max_ = 445 nm) and R, red LED light (λ_max_ = 660 nm). The light intensity of each spectrum was maintained at 200 ± 10 μmol m^−2^ s^−1^ PPFD with 16 h photoperiod. Leaf samples were collected for plant metabolite analyses and *in vitro* anti-*Plasmodium falciparum* assay with (3 x 3) x 2 experimental replications per treatment.

### Determination of artemisinin and artemisinic acid by HPLC

Artemisinin was extracted following the modified protocol of Banyai et al. [45]. Briefly, fresh leaves were immersed in 10 mL dichloromethane and vortexed for 1 min. The leaves were removed and the solvent was allowed to completely evaporate in a fume hood. Quantitation of artemisinin was carried out using the Q260 assay as the standard method for artemisinin determination used in several recent studies [72–74]. Artemisinin derivatization was performed following the modified protocol of Vandenberghe et al. [75]. First, the crude extract was re-dissolved in 1 mL methanol. Then, 200 μL of the extract was mixed with 800 μL of 0.2% (w/v) sodium hydroxide. After vortexing, the mixture was incubated in a 50 °C water bath for 30 min. Methanol containing 0.05 M acetic acid was added into the cooled reaction mixture at a 1:4 ratio. The mixture was then filtered through a 0.45 μm Sartorius® membrane before injection. Artemisinin was quantified by HPLC with a UV detector at a wavelength of 260 nm (Waters 2690, USA) using a Luna 5 μm C18 100 Å column (250 mm x 4.6 mm^2^; Phenomenex, USA). A mixture of 55% (v/v) acetonitrile in water containing 0.05% (v/v) formic acid was used as the mobile phase. Authentic artemisinin powder (Kunming Pharmaceutical, China) was dissolved in methanol and its serial dilutions were used to construct a standard curve.

For artemisinic acid measurement, approximately 0.5 g of oven-dried *A. annua* leaves were ground into a fine powder using a sterile mortar and pestle. The extract was obtained by mixing the powder with methanol and filtering through a 0.45 μm Sartorius® membrane. The filtrate was analyzed by HPLC with a UV detector at a wavelength of 192 nm, as suggested by Ferreira and Gonzalez [76]. The mobile phase was composed of 60% (v/v) acetonitrile and 40% (v/v) of water containing 0.1% acetic acid (pH 3.2) with a flow rate of 1.0 mL min^−1^. Standard artemisinic acid (Wuhan ChemFaces Biochemical, China) was dissolved in methanol and serially diluted to construct the standard curve.

### Determination of terpenoid compounds by GC-MS

To prepare the extract, 50 mg of dried leaf powder was suspended in 1 mL dichloromethane and sonicated at room temperature for 15 min, before filtering through a 0.45 μm filter membrane (Millipore, USA). The extracts were kept at 4 °C until required for analysis. GC-MS analysis was performed according to the previously reported protocol [52] using an Agilent 6890 N network gas chromatograph system (Agilent Technologies Inc., USA) equipped with HP-5 ms GC capillary columns (5% diphenyl 95% dimethylpolysiloxane, 30 m × 0.25 mm, film thickness 0.25 μm) and a 5973 N network mass selective detector (Agilent Technologies Inc., USA). Relative abundance of the compounds was quantified by comparing their peak areas with the internal standard (methyl heptadecanoate, C17) and represented as a relative content (%) [37, 52, 77]. Mass spectral information of the peaks was compared with the mass spectral library (Wiley 7 no. 1) to identify the compounds. Only compounds with at least 70% match quality were reported and further analyzed.

### *In vitro* anti-*Plasmodium falciparum* assay

Leaf extracts from different light treatments were used for growth inhibition analysis of the standard laboratory strain (NF54) of *Plasmodium falciparum*, with the culture maintained at the Malaria Unit, Department of Pathobiology, Faculty of Science, Mahidol University. An *in vitro* culture of *P. falciparum* NF54 was synchronized by 5% sorbitol (PanReac AppliChem ITW Reagents, Germany) to prepare a ring stage culture [78]. This method resulted in selective destruction of trophozoite- and schizont-infected red blood cells, as sorbitol only penetrated the membrane of cells harboring these stages of the parasite, leading to their osmotic lysis. A 1% (v/v) of ring stage culture was co-cultured with various concentrations of leaf extracts, ranging from 0.001 to 100 μg mL^−1^. Artesunate (100 nM) (Guilin Pharmaceutical, China) was used as the positive control, while 0.05% DMSO was used as the negative control. After 28–30 h of incubation, blood smears of the extract-treated parasite cultures were prepared and stained with Giemsa stain (VWR®, Germany). The percentage of parasitemia and morphological changes were observed under a light microscope BX53 (Olympus, Japan). The percentage of growth inhibition was calculated by comparison with the untreated group. The IC_50_ values were determined by Probit analysis (IBM® SPSS® Statistics 24).

### Clustering analysis of plant metabolites under light treatments and antimalarial activity

To examine the correlation between light treatments and compound abundance and bioactivity, HCA was performed using a MultiExperiment Viewer (MeV) version 4.9.0. Quantitative data of bioactive compounds were obtained from HPLC, while the relative contents of terpenoid compounds were obtained from GC-MS data, normalized to the amount of the internal standard. Grouping results were used to construct a dendrogram, with a heatmap representing the content of each compound. Datasets obtained from the experiments were clustered and grouped based on similarity. Relative branch lengths of the dendrograms indicated differences between the expression of each treatment or compound.

### Statistical Analysis

Data were analyzed using PASW Statistics 18.0.0. Results were expressed as mean (± SD). One-way ANOVA, followed by Tukey’s HSD test, was used to determine the statistical differences between the results of leaf FW, bioactive compound measurements, and the growth inhibition of *P. falciparum* NF54.

## Supplementary Information

**Additional file 1.**

## Data Availability

Not applicable.

## Abbreviations

ATS: Artesunate
B: Blue LED light
DMSO: Dimethyl sulfoxide
FW: Fresh weight
GC-MS: Gas chromatography-mass spectrometry
GST: Glandular secretory trichome
HCA: Hierarchical clustering analysis
HPLC: High-performance liquid chromatography
LED: Light-emitting diode
PFAL: Plant factory with artificial lighting
PPFD: Photosynthetic photon flux density
R: Red LED light
RH: Relative humidity
W: White LED light
