# Supplementary material for: Variation in terpenoids in leaves of Artemisia annua grown under different LED spectra resulting in diverse antimalarial activities against Plasmodium falciparum
Source: BMC Plant Biol. 2022 Mar 21;22:128. doi: 10.1186/s12870-022-03528-6 (PMC8935710; doi:10.1186/s12870-022-03528-6)
Supplement: Supplementary file 1 — Additional file 1. [file 12870_2022_3528_MOESM1_ESM.docx]

**Variation in terpenoids in leaves of *Artemisia annua* grown under different LED spectra resulting in diverse antimalarial activities**

**against *Plasmodium falciparum***

Darunmas Sankhuan^a^, Gamolthip Niramolyanun^b^, Niwat Kangwanrangsan^b^,

Masaru Nakano^c^, Kanyaratt Supaibulwatana^a*^

*^a^Department of Biotechnology, Faculty of Science, Mahidol University, 272 Rama VI Road, Ratchathewi District, Bangkok, 10400, Thailand*

*^b^Department of Pathobiology, Faculty of Science, Mahidol University, 272 Rama VI Road, Ratchathewi District, Bangkok, 10400, Thailand*

*^c^Faculty of Agriculture, Niigata University, 2-8050, Ikarashi, Niigata, 9502181, Japan*

*** Corresponding author:**

Kanyaratt Supaibulwatana: at Department of Biotechnology, Faculty of Science, Mahidol University, 272 Rama VI Road, Ratchathewi District, Bangkok, 10400, Thailand

*E-mail address:* [kanyaratt.sup@mahidol.ac.th](mailto:kanyaratt.sup@mahidol.ac.th) (K. Supaibulwatana)

**Supplementary Information**

**Table S1** Leaf fresh weights, contents, yields of artemisinin and artemisinic acid and antimalarial activity from leaf extracts of *A. annua* plants that grown in greenhouse

| **Treatment** | **Greenhouse** ^a^ |
| --- | --- |
| Leaf FW (g) | 1.02 ± 0.02 |
| Artemisinin content (mg mL^-1^ dry weight) | 0.43 ± 0.00 |
| Artemisinic acid content (mg mL^-1^ dry weight) | 0.15 ± 0.01 |
| Artemisinin yield (mg plant^-1^) | 0.44 ± 0.01 |
| Artemisinic acid yield (mg plant^-1^) | 0.16 ± 0.01 |
| IC_50_ against *P. falciparum* NF54 (µg mL^-1^) ^b^ | 2.23 |

^a^ The 45-day-old *A. annua* plants were incubated in the greenhouse under natural light (N) for 7 days. The average microclimate condition during 17-23 January 2019 was recorded as 38.5 ± 0.5 °C, and 59.4 ± 1.7% RH. The report from Meteorological Department of Thailand and The Thai Astronomical Society indicated the average light intensity at 1,045 ± 35 μmol m^-2^ s^-1^ PPF with an average 11.4 h of photoperiod.

^b^ The IC_50_ values were determined by Probit analysis (IBM® SPSS® Statistics 24)

**Table S2** Phytochemical profile of leaf extracts of *A. annua* grown under different conditions for 7 days. The metabolites were determined by GC-MS and quantified by comparing with the internal standard (methyl heptadecanoate, C17). Only compounds with more than 70% match quality to the Wiley No.7 database were shown.

| **Compound type** | **RT** | **Compound name** | **% Relative content** | | | |
| --- | --- | --- | --- | --- | --- | --- |
|  |  |  | **N** | **W** | **B** | **R** |
| Non-terpenes | 25.33 | Coumarin | 27.9 | 6.0 | - | - |
|  | 26.32 | 1-Dodecene | - | - | - | 7.4 |
|  | 27.52 | 2,4-Di-tert-butylphenol | - | - | - | 3.3 |
|  | 32.68 | Dodecyl acrylate | - | - | - | 9.9 |
|  | 38.73 | 3-methyl-7-methoxy-2-benzopyran-1(1H)-one | 12.3 | 25.3 | 32.3 | 22.5 |
|  | 39.70 | Scopoletin | 6.1 | 13.4 | 16.3 | 19.1 |
|  | 39.97 | 3a,9-Dimethyldodecahydrocyclohepta[d] inden-3-one | 4.0 | - | 12.0 | - |
|  | 40.79 | Propanoic acid, 3-mercapto-, dodecyl ester | - | - | - | 6.1 |
|  | 45.00 | Docosane | 1.3 | - | - | - |
|  | 47.13 | Tricosane | 4.7 | 5.4 | - | - |
|  | 49.19 | Tetracosane | 6.6 | 7.9 | - | 2.9 |
|  | 50.65 | (6Z,8E)-6,8-dodecadien-1-ol | 2.1 | 6.6 | 3.9 | - |
|  | 51.00 | 1-Octadecanol | 2.3 | 4.1 | 3.3 | 2.9 |
|  | 51.18 | Pentacosane | 10.0 | 9.7 | - | - |
|  | 51.38 | Hexadecanoic acid, 2-hydroxy-1-(hydroxymethyl)ethyl ester | 7.5 | 7.1 | 8.0 | - |
|  | 53.09 | Nonacosane | 10.6 | 11.0 | - | - |
|  | 54.75 | Beta-Monolinolein | 4.8 | 6.8 | 5.8 | 5.2 |
|  | 54.87 | 1-Docosene | 23.0 | 42.3 | 46.5 | 34.9 |
|  | 54.95 | Hexadecane | 15.4 | 15.2 | - | - |
|  | 56.67 | 1-Dotriacontanol | 2.0 | 13.7 | 3.7 | 4.3 |
|  | 56.71 | Octacosane | 12.3 | - | - | - |
|  | 58.52 | Cyclotetracosane | 62.8 | 109.9 | 98.4 | 75.3 |
|  | 59.24 | 1-Hexacosanol | 2.1 | - | 4.1 | 3.2 |
|  | 60.10 | Tetradecane | 10.5 | 10.6 | 4.6 | 3.7 |
|  | 61.84 | 1-Hexacosanol | 58.1 | 85.1 | 76.4 | 66.5 |
|  | 62.31 | D,.alpha.-Tocopherol | 3.8 | 11.7 | 17.7 | 8.9 |
|  | 62.54 | Z-14-Nonacosane | 1.9 | - | 3.0 | 2.4 |
|  | 62.69 | 2,4-Dimethylhexane | - | - | - | 8.0 |
|  | 63.29 | Dotriacontane | 3.2 | - | - | - |
|  | 64.79 | Hexadecane | 2.6 | - | - | - |
|  | 64.92 | 1-Eicosanol | 6.9 | 11.3 | 10.7 | 9.3 |
|  | 65.58 | 2,5-Dimethylhydroquinone | - | - | - | 3.5 |
|  | 67.98 | 2-Pentacosanone | 2.6 | - | - | - |
| Monoterpenes | 8.50 | Camphene | - | - | 4.9 | 3.5 |
|  | 11.31 | Eucalyptol | - | - | 4.7 | 4.4 |
|  | 15.36 | Camphor | 30.0 | 62.8 | 69.8 | 63.7 |
|  | 16.08 | Borneol | 2.7 | 6.4 | 12.4 | 7.5 |
|  | 17.18 | 2-Methylnorbornene | 4.4 | - | 4.7 | 4.7 |
|  | 20.43 | Nerol | - | 5.9 | 4.1 | 3.8 |
|  | 21.94 | Santolina triene | 1.6 | 6.1 | 6.2 | 6.8 |
| Sesquiterpenes | 23.22 | Alpha-copaene | 2.5 | 5.9 | 5.4 | 5.7 |
|  | 24.62 | Trans-Caryophyllene (I) | 16.3 | 61.1 | 60.3 | 43.7 |
|  | 24.94 | Beta-Cubebene | 3.0 | 9.9 | 9.1 | 7.7 |
|  | 25.41 | Germacrene D (I) | 3.1 | 6.6 | 6.1 | 4.2 |
|  | 25.69 | Alpha-Humulene | 2.2 | 7.9 | 7.2 | 6.1 |
|  | 25.81 | Trans-beta-Farnesene | 29.5 | 54.8 | 56.6 | 104.1 |
|  | 26.39 | Alpha-Neoclovene | - | 7.1 | - | 4.4 |
|  | 26.60 | Germacrene D (II) | 30.2 | 120.1 | 88.1 | 78.8 |
|  | 26.75 | Beta-Selinene | - | 3.8 | - | - |
|  | 27.03 | Germacrene B | 2.5 | 12.5 | 10.1 | 11.2 |
|  | 29.34 | Trans-Caryophyllene (II) | - | 7.5 | - | - |
|  | 32.46 | Alpha-Bisabolol | - | 6.3 | - | - |
|  | 41.64 | Deoxyqinghaosu | 17.5 | 36.9 | 36.3 | 33.0 |
| Diterpenes | 36.53 | Neophytadiene (I) | 22.9 | 42.8 | 36.4 | 24.3 |
|  | 36.68 | 1-Phytene | - | 6.3 | 5.2 | - |
|  | 37.12 | Neophytadiene (II) | 3.5 | 5.4 | 4.8 | 3.6 |
|  | 37.58 | Neophytadiene (III) | 7.5 | 7.2 | 9.3 | 8.2 |
|  | 43.18 | Phytol | 71.1 | 84.4 | 55.4 | 57.2 |
|  | 68.38 | Neophytadiene (IV) | - | 9.6 | 19.2 | 19.7 |
| Triterpenes | 57.25 | Squalene | 10.2 | 8.0 | 8.7 | 30.4 |
|  | 63.71 | Ergost-5-en-3-beta-ol | 3.1 | 6.4 | 5.2 | 5.4 |
|  | 64.26 | Stigmasta-5,22-dien-3-ol | 22.9 | 39.7 | 32.3 | 35.2 |
|  | 65.13 | Gamma-Sitosterol | 10.1 | 21.4 | 22.9 | 19.0 |
|  | 65.29 | Urs-12-en-3-one | 23.5 | 34.5 | 27.5 | 21.1 |
|  | 65.91 | 3-Keto-urs-12-ene | 38.6 | 72.3 | 44.8 | 61.3 |
|  | 66.27 | Alpha-Amyrin | 9.5 | 17.3 | 11.8 | 16.3 |
|  | 66.64 | Isomultiflorenone | - | 6.9 | - | - |
|  | 66.92 | Lupeol | 3.2 | 15.2 | 4.9 | 6.0 |

N; leaf extract from the greenhouse under natural light, W; leaf extract from PFAL with white spectrum (445, 554 nm), B; leaf extract from PFAL with blue spectrum (445 nm), R; leaf extract from PFAL with red spectrum (660 nm)


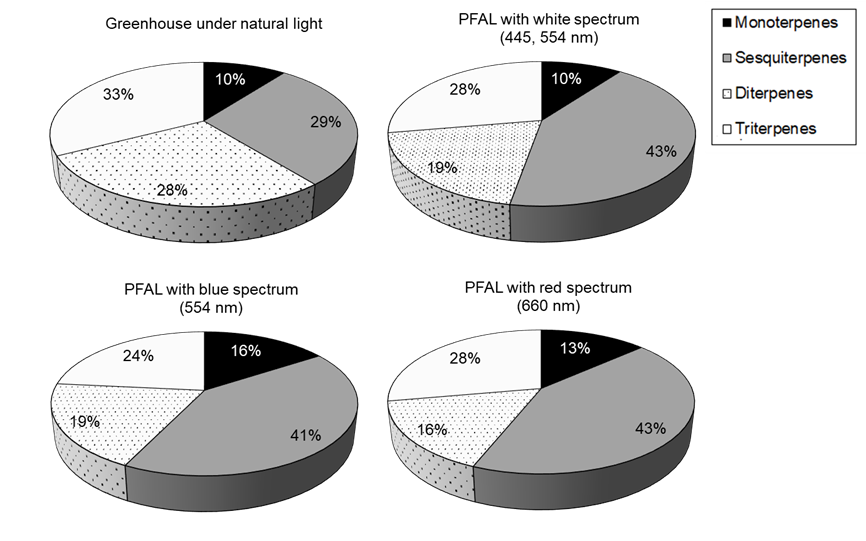


**Fig. S1** Terpenoid compositions of leaf extracts derived from *A. annua* grown under different conditions for 7 days.
